# Targeting the oncogenic protein beta-catenin to enhance chemotherapy outcome against solid human cancers

**DOI:** 10.1186/1476-4598-9-310

**Published:** 2010-12-02

**Authors:** Maher S Saifo, Donald R Rempinski, Youcef M Rustum, Rami G Azrak

**Affiliations:** 1 Department of Cancer Biology, Roswell Park Cancer Institute, Buffalo, New York, USA; 2 Department of Oncology, Albairouni University Hospital, Damascus University, Damascus Syria

## Abstract

**Background:**

Beta-catenin is a multifunctional oncogenic protein that contributes fundamentally to cell development and biology. Elevation in expression and activity of β-catenin has been implicated in many cancers and associated with poor prognosis. Beta-catenin is degraded in the cytoplasm by glycogen synthase kinase 3 beta (GSK-3β) through phosphorylation. Cell growth and proliferation is associated with β-catenin translocation from the cytoplasm into the nucleus.

This laboratory was the first to demonstrate that selenium-containing compounds can enhance the efficacy and cytotoxicity of anticancer drugs in several preclinical xenograft models. These data provided the basis to identify mechanism of selenium action focusing on β-catenin as a target. This study was designed to: (1) determine whether pharmacological doses of methylseleninic acid (MSeA) have inhibitory effects on the level and the oncogenic activity of β-catenin, (2) investigate the kinetics and the mechanism of β-catenin inhibition, and (3) confirm that inhibition of β-catenin would lead to enhanced cytotoxicity of standard chemotherapeutic drugs.

**Results:**

In six human cancer cell lines, the inhibition of total and nuclear expression of β-catenin by MSeA was dose and time dependent. The involvement of GSK-3β in the degradation of β-catenin was cell type dependent (GSK-3β-dependent in HT-29, whereas GSK-3β-independent in HCT-8). However, the pronounced inhibition of β-catenin by MSeA was independent of various drug treatments and was not reversed after combination therapy.

Knockout of β-catenin by ShRNA and its inhibition by MSeA yielded similar enhancement of cytotoxicity of anticancer drugs.

Collectively, the generated data demonstrate that β-catenin is a target of MSeA and its inhibition resulted in enhanced cytotoxicity of chemotherapeutic drugs.

**Conclusions:**

This study demonstrates that β-catenin, a molecule associated with drug resistance, is a target of selenium and its inhibition is associated with increased multiple drugs cytotoxicity in various human cancers. Further, degradation of β-catenin by GSK-3β is not a general mechanism but is cell type dependent.

## Background

Beta-catenin protein is a vital component of the canonical Wnt/β-catenin signaling pathway, which is described as an oncogenic cause in many human cancers [1]. In head and neck squamous cell carcinomas (HNSCC), over expression of the Wnt/β-catenin signaling pathway increases cell survival and invasion [2]. The higher β-catenin expression in HNSCC patients, the more advanced stage [3] and poor prognosis are observed [4]. Mutations in the gene that encodes β-catenin (CTNNB1) [5] and elevated nuclear β-catenin [6] were implicated in prostate cancers (CaP).

Over 90% of colorectal cancers (CRC) demonstrate a deregulated Wnt/β-catenin signaling pathway [7]. Published studies suggest that unregulated β-catenin, overlapping with adenomatous polyposis coli (APC) mutation, is associated with the initiation of CRC [8-10]. Beta-catenin is expressed in the cytoplasm and the nucleus. The cytoplasm β-catenin, as a component of adherens junctions (AJs) [11], is an essential element of cell-to-cell adhesion and stability. The level of cytoplasm β-catenin is controlled by the activity of a destruction complex that consists of axin, glycogen synthase kinase 3β (GSK-3β) and APC [12-15]. In the absence of Wnt signaling, the complex is assembled and GSK-3β phosphorylates and consequently degrades cytoplasm β-catenin [14,15]. However, GSK-3β is inactivated in cancer cells by phosphorylation at serine 9, a similar mechanism of GSK-3β inhibition by lithium [16,17]. In the presence of Wnt signaling, β-catenin destruction complex is disassembled by removing axin [18,19] resulting in β-catenin accumulation in the cytoplasm. The accumulated cytoplasm β-catenin hence enters the nucleus to initiate its oncogenic function. The nuclear β-catenin has an important function in many human malignancies [1] by stimulating cell growth and proliferation. The nuclear β-catenin affects TCF/LEF family transcription factors [20,21] and consequently activates oncogenes such as cyclin D1 [22,23], Myc [24] and many other downstream targets. The nuclear accumulation of β-catenin is a critical step in the activation process of the canonical Wnt signaling pathway and is associated with poor prognosis in cancer patients [25].

In addition to its role in cell growth and adhesion, activated canonical Wnt/β-catenin signaling pathway is linked to cancer stem cells [26,27] that contribute to tumor bulk, recurrence and resistance to chemotherapy. Accordingly, β-catenin inhibitors in combination with standard systemic therapies hold great promise to improve treatment's efficacy and outcome.

The response rates of combination regimen of irinotecan and 5-fluorouracil/leucovorin (5-FU/LV) is 39% in metastatic CRC [28]. Treatment with oxaliplatin and 5-FU/LV has improved the response rate to 50.7% in CRC [29]. Treatment with docetaxel and prednisone against metastatic CaP resulted in a median survival of 19.2 months [30]. Docetaxel in combination with cisplatin and 5-FU against inoperable advanced HNSCC resulted in a median progression free survival of 11 months [31]. Although the relative survival in advanced solid tumors is improved by using systemic therapy, the current chemotherapy cure rates are limited. Thus, the development of new regimens is greatly needed to achieve a better clinical outcome.

In our preclinical models, selenium-containing compounds enhanced the efficacy of multiple chemotherapeutic agents (CPT-11 or docetaxel) against various types of cancers (colorectal, head and neck and prostate) [32,33]. In mice bearing human colorectal cancer xenografts (HCT-8), combination treatment of MSC and irinotecan resulted in complete tumor regression (100% CR) that was not observed with each drug alone (30% CR) [32]. Sequential combination of MSeA and docetaxel resulted in synergy enhancing docetaxel-induced cell death in CaP [33]. Multiple mechanisms of the synergy between selenium and other chemotherapeutic agents are proposed. Selenium (Se) is an essential element that possesses antioxidant properties in a form of selenoproteins protecting cells from harmful free radicals [34-36]. The effect of selenium on β-catenin has yet to be investigated.

This study is designed mainly to determine if β-catenin is a target of MSeA in CRC, HNSCC and CaP cancers; and to evaluate the role of GSK-3β in the degradation of β-catenin and if such an effect is associated with enhanced cytotoxicity of anticancer drugs.

## Results

### Inhibition of β-catenin by MSeA is concentration, time and tumor type dependent

**Figure 1:**
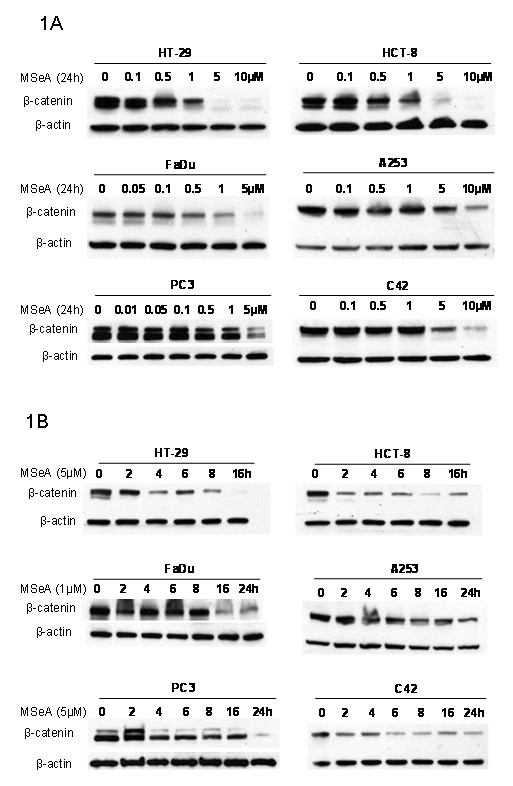
**MSeA effect on intracellular expression of β-catenin**. Six human cancer cell lines including 2 colorectal (HT-29 and HCT-8), 2 head and neck (FaDu and A253) and 2 prostate (PC3 and C42) were treated with various doses of MSeA (panel 1A) and various times (panel 1B). Western blot analyses show that MSeA inhibits intracellular level of β-catenin in dose and time dependent manners.

To evaluate the effect of MSeA on the expression of β-catenin, various tumor cell types were treated with multiple time and doses of MSeA. In all treated cells, MSeA decreased the expression of β-catenin in dose and time dependent manners (Figure 1). The data in Figure 1 indicate that the down regulation of β-catenin is MSeA concentration dependent. In CRC cells (HCT-8 and HT-29), 24 h treatment with 5 μM resulted in completely depletion of β-catenin. In contrast, in HNSCC cells (FaDu and A253), the decrease in β-catenin levels in FaDu cells was achieved with lower concentrations of MSeA (0.5 μM) than in A253 (5 μM) (Figure 1A). In the androgen-independent CaP cells (PC3 and C42), inhibition of β-catenin by MSeA required a high concentration (5 μM) (Figure 1A). The kinetics of β-catenin inhibition by MSeA appears to be tumor type dependent, early in HCT-8, C42, HT-29 and PC3, and late event in FaDu and A253 (Figure 1B).

### MSeA inhibits β-catenin nuclear expression

**Figure 2:**
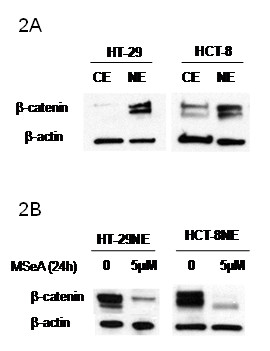
**MSeA effect on the nuclear expression of β-catenin**. Colorectal cancer cells express a high-level of nuclear β-catenin indicating activation (panel 2A). Treatment with MSeA results in inhibition of the nuclear expression of β-catenin indicating suppression of the active form of β-catenin (panel 2B).

To determine whether MSeA down regulates the activity of β-catenin, nuclear and cytoplasmic extracts of colorectal cancer cells were tested for the level of β-catenin before and after MSeA treatment. The data in Figure 2A indicate that β-catenin is predominantly expressed in the nucleus of untreated CRC cells (HCT-8 and HT-29) indicating activation of β-catenin. Treatments with MSeA resulted in inhibition of nuclear expression of β-catenin (Figure 2B). These data suggest that MSeA down regulates β-catenin activity through inhibiting its nuclear expression.

### Inhibition of β-catenin is due to enhanced degradation

**Figure 3:**
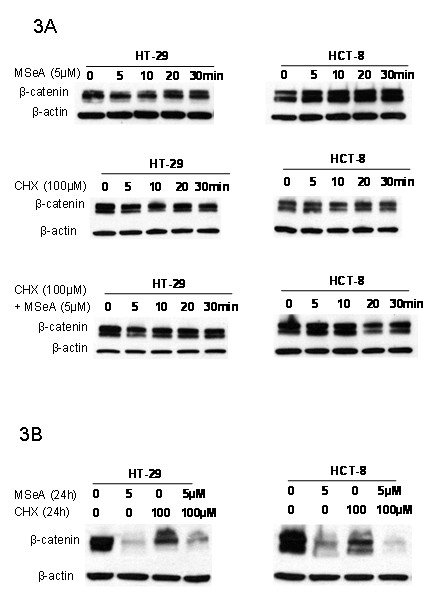
**MSeA inhibition of β-catenin is due to degradation**. Colorectal cancer cells were treated with a protein synthesis inhibitor, cycloheximide (CHX) and MSeA alone or in combination for various times up to 30 minutes (panel 3A) and for 24 h (panel 3B). No early changes in the β-catenin level were observed (panel 3A). However, 24 h combination treatment with CHX/MSeA resulted in same inhibition after MSeA alone (panel 3B).

To determine whether the observed down regulation of β-catenin by MSeA results from inhibition of its synthesis or from increased degradation, cells were treated with MSeA and cycloheximide (an inhibitor of de novo protein synthesis [37]) alone and in combination. Treatments for up to 30 minutes did not affect the expression level of β-catenin (Figure 3A). However, β-catenin is down regulated after 24 h treatment with MSeA alone and in combination with cycloheximide (Figure 3B). These data suggest that the inhibition of β-catenin by MSeA is the result of increased degradation.

### The role of GSK-3β in β-catenin degradation is cell type dependent

**Figure 4:**
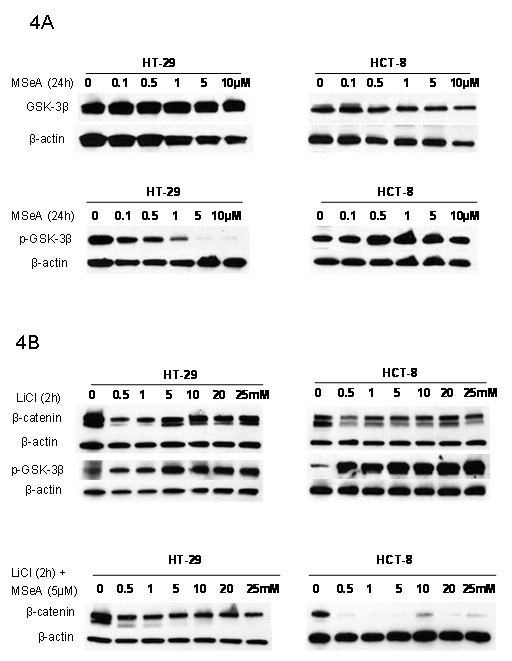
**The role of GSK-3β in degradation of β-catenin**. Colorectal cancer cells were treated with various doses of MSeA alone (panel 4A) or in combination with GSK-3β inhibitor, Lithium Chloride (LiCl, panel 4B). The level of total GSK-3β, p-GSK-3β and β-catenin were determined using western blots. Treatment with MSeA alone had no effect on total GSK-3β but decreased the level of p-GSK-3β only in HT-29 (panel 4A). Treatments with LiCl increased the level of p-GSK-3β (inactive form of GSK-3β) but had no effect on β-catenin expression (panel 4B). Combination treatment of LiCl/MSeA continued to inhibit expression of β-catenin only in HCT-8 (panel 4A)

To determine the mechanism of β-catenin degradation by MSeA, the role of GSK-3β was evaluated in HT-29 and HCT-8 (Figure 4). Treatments with MSeA had no significant effect on the level of total GSK-3β (in HT-29 or HCT-8) and on the level of phosphorylated GSK-3β in HCT-8. In contrast, phosphorylated GSK-3β was significantly decreased in HT-29 (Figure 4A).

To evaluate further the role of GSK-3β, cells were treated with lithium chloride (LiCl) alone (GSK-3β inhibitor [38]) and in combination with MSeA. Treatment with LiCl increased the level of phosphorylated GSK-3β in both cell lines indicating inhibition of GSK-3β (Figure 4B). Combination of MSeA with various doses of LiCl resulted in reversing the down regulation of β-catenin in HT-29 cells but not HCT-8 by MSeA (Figure 4B). Data in Figure 4 demonstrated that the inhibition of β-catenin by MSeA is GSK-3β phosphorylation dependent in HT-29 but independent in HCT-8.

### The effect of MSeA in combination therapy on the level and activity of β-catenin

**Figure 5:**
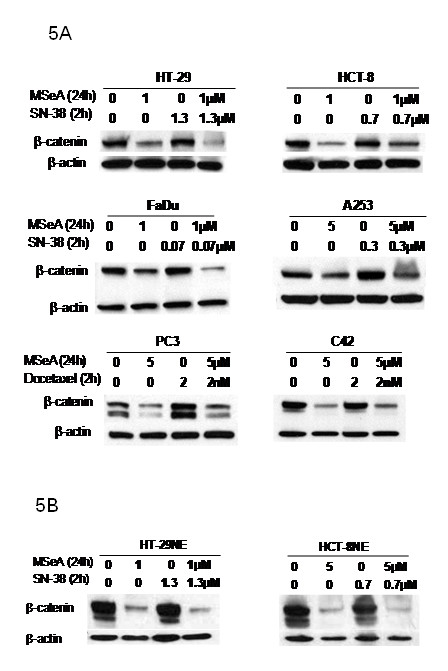
**Combination treatment effect on the expression of β-catenin**. Cancer cells (panel 5A) and nuclear extraction of colorectal cancer cells (panel 5B) were treated with MSeA, SN-38 and docetaxel alone or in combination. Expression of β-catenin was determined using western blots. Combination treatments of MSeA/SN-38 continued to decrease expression of β-catenin when compared with all other groups. Combination treatments of MSeA/docetaxel did not reverse the inhibitory effect of MSeA alone on expression of β-catenin (panel 5A). The active form (nuclear expression) of β-catenin was inhibited by the combination treatment of MSeA/SN-38 when compared with other group (panel 5B).

To determine whether MSeA in combination with a chemotherapeutic agent would affect β-catenin expression and activity, cells were treated with MSeA ± chemotherapeutic agent and analyzed for total and nuclear β-catenin expression. The data in Figure 5A indicate that MSeA inhibited β-catenin expression in all cell lines but neither 7-Ethyl-10-Hydroxycamptothecin (SN-38, the active metabolite of irinotecan) nor docetaxel alone had an effect on the β-catenin levels. Adding SN-38 or docetaxel to MSeA did not interfere with selenium inhibition of β-catenin. The combination of MSeA/SN-38 resulted in even more observed inhibition of total β-catenin when compared with MSeA alone in HT-29, HCT-8 and FaDu cells. In CaP cells, MSeA alone and in combination with docetaxel have similar inhibitory effect on the expression of β-catenin (Figure 5A). To determine whether the inhibition of β-catenin by MSeA in combination with SN-38 is due to inhibition of the nuclear expression, nuclear extracts of CRC cells were treated with MSeA alone and in combination with SN-38 and evaluated for the level of β-catenin. The combination treatment of MSeA/SN-38 resulted in down regulation of the nuclear expression of β-catenin in HT-29 and HCT-8 cells (Figure 5B). These results indicate that the activity of β-catenin is decreased after the combination therapy.

### The inhibition of β-catenin expression by ShRNA or MSeA is associated with enhancement of drug-induced growth inhibition of tumor cell

**Figure 6:**
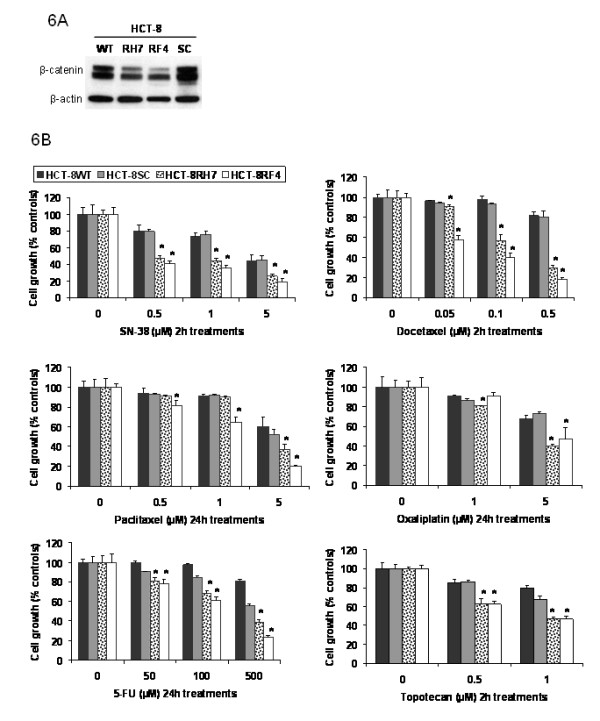
**The effect of various drug treatments on cell growth and proliferation in β-catenin knockout cells**. Beta-catenin was silenced in colorectal cancer cells (HCT-8, panel 6A). HCT-8 wild type cells (HCT-8WT), scramble controls (HCT-8SC) and 2 β-catenin knockout clones (HCT-8RH7 and HCT-8RF4) were tested. Expression of β-catenin was significantly lower in HCT-8RH1 and HCT-8RF4 when compared with all other groups (panel 6A). Cancer cell growth and proliferation were determined in HCT-8WT, HCT-8SC, HCT-8RH7 and HCT-8RF4 after treatment with various doses of SN-38, docetaxel, paclitaxel, oxaliplatin, 5-FU and topotecan using SRB assay. HCT-8RH7 and HCT-RF4 cells were significantly more sensitive to various drug treatments when compared with HCT-8WT and HCT-8SC (panel 6B). * denotes a p value of less than 0.05 when compared with all other groups.

To determine whether inhibition of β-catenin can be correlated with enhanced efficacy of chemotherapy, β-catenin in tumor cells was knockdown by specific ShRNA. Two β-catenin ShRNA tranfectant clones (HCT-8RH7 and HCT-8RF4) that demonstrated inhibition of β-catenin expression when compared with scrambled control (HCT-8SC) and wild type (HCT-8WT) were selected for further testing (Figure 6A).

To evaluate the effect of silencing β-catenin on cell growth, HCT-8WT, HCT-8SC and HCT-8R (HCT-8RH7 and HCT-8RF4) transfectants were treated with various classes of chemotherapeutic agents. Treatments with 0.5 μM of SN-38 were more effective (p < 0.05) against HCT-8R (~50% cell growth inhibition) when compared with all other groups (15% cell growth inhibition in HCT-8SC or HCT-8WT, Figure 6B). Other doses of SN-38 resulted in similar patterns of inhibition of cell growth (Figure 6B). In similar fashion, treatments with various doses of docetaxel, paclitaxel, oxaliplatin, 5-FU and topotecan significantly enhanced efficacy against cell growth of HCT-8RH7 and HCT-8RF4 when compared with all other groups of HCT-8SC or HCT-8WT (Figure 6B).

To confirm further that inhibition of β-catenin is associated with enhanced cytotoxicity of anticancer drugs, tumor cells were treated with MSeA alone and in combination with SN-38 and the results were correlated with the levels of β-catenin (table 1). The data in table 1 demonstrated a relationship between enhanced cytotoxicity of SN-38 and inhibition of β-catenin by MSeA or ShRNA. Thus, these data support the initial hypothesis that inhibition of β-catenin by MSeA is a critical determinant of drug response.

**Table 1:** The inhibition of β-catenin expression by ShRNA or MSeA is associated with drug-induced growth inhibition of tumor cells

HCT-8 cell line	% cell growth inhibition	Beta-catenin expression
Control	0%	++++/strong
MSeA (1 μM)	~50%	++/weak
SN-38(0.7 μM)	~20%	++++/strong
MSeA+SN-38	>95%	+/very weak
ShRNA	0%	++/weak
ShRNA+SN38	~70%	+/very weak

## Discussion

Beta-catenin oncogonic protein is widely expressed in many human malignancies [1] including HNSCC [2-4], CaP [5,6] and CRC [8-10]. Beta-catenin is involved in cell growth [22-24], adhesion [11] and stemness [26,27]. Beta-catenin is found in multiple cellular locations including intracellular membrane, cytoplasm and nucleus. The nuclear accumulation of β-catenin indicates the activation of its oncogenic form that stimulates transcription factors and genes [22-24] leading to enhanced tumor cell growth and poor prognosis [25]. The hypothesis of this study is that β-catenin is a target of MSeA and its inhibition would translate into enhanced drug effect. Our results (Figure 1) established that MSeA is a potent inhibitor of β-catenin in various cancer types. This broad inhibitory effect of MSeA on the expression of β-catenin is pivotal for explaining the established synergy between selenium and various chemotherapeutic agents against multiple cancers.

The data in Figure 2 demonstrate that the inhibition of β-catenin level after MSeA is due to the inhibition of the active form of β-catenin in the nucleus. Thus, these data indicate that pharmacologic doses of MSeA offer effective inhibition of β-catenin activation.

Recent findings by Zhang et al demonstrate that selenium effect against esophageal squamous cell carcinoma is correlated with its inhibition on β-catenin/TCF pathway [39]. Our results confirm this finding in various human cancers and prove that the decreased level of β-catenin is associated with enhanced efficacy of various classes of chemotherapy. Thus, indicating the importance of β-catenin inhibition in drug response.

To determine whether the inhibition of β-catenin is due to a decrease in synthesis or an increase in degradation, de novo protein synthesis was inhibited using CHX in the presence and absences of MSeA. The data in Figure 3 indicate that MSeA inhibition of β-catenin is due to increase in degradation but not decrease in synthesis in both CRC cells (HT-29 and HCT-8). Many studies have showed that cytoplasm β-catenin is degraded by an axin/GSK-3β/APC complex [12-14] and the degradation is a GSK-3β phosphorylation dependent process [14,15]. The degradation of the cytoplasm β-catenin prevents its accumulation and translocation into the nucleus. Data in Figure 4 demonstrated that the inhibition of β-catenin by MSeA is GSK-3β dependent phosphorylation in HT-29 but independent in HCT-8. The GSK-3β independent degradation of β-catenin is a novel finding in HCT-8 cells and indicates that the MSeA effect involves other signaling pathways than Wnt/β-catenin, which will be investigated in future studies.

In preclinical models, sequential combination treatment of selenium compounds (MSC, SLM or MSeA) and various chemotherapeutic agents (SN-38 or docetaxel) were proven synergistic against various cancers including HNSCC, CRC and CaP [32,33]. Studies were carried out to determine whether the combination treatment of MSeA and chemotherapeutic agents affect β-catenin level in those cell lines. Our results in Figure 5 showed that treatment with MSeA in combination with SN-38 or docetaxel down regulated the total and the nuclear β-catenin. These results confirm that the chemotherapeutic agent did not interfere with selenium inhibition of the level and activity of β-catenin. However, neither SN-38 nor docetaxel alone affect the expression level of β-catenin (Figure 5).

To determine that inhibition of β-catenin by MSeA will translate into enhanced drug-cytotoxicity, cells knocked down β-catenin by ShRNA were more sensitive to growth inhibition by SN-38 than wild type. Collectively, this study indicates that the decreased level of β-catenin is associated with enhancement of drug induced inhibition of cell growth. (table 1).

Further, the data in Figure 6 indicate that silencing β-catenin increases the cytotoxicity of various chemotherapeutic agents. The efficacy of SN-38, docetaxel, paclitaxel, oxaliplatin, 5-FU and topotecan was significantly increased in HCT-8R when compared with control groups (Figure 6B).

## Conclusions

These results support the hypothesis that β-catenin is a target of MSeA and its inhibition results in enhanced drug-cytotoxicity in multiple cancers. Degradation of β-catenin by GSK-3β is not a general mechanism but it is cell type dependent.

Although Selenium is a multi-target agent [32,33,40], inhibition of β-catenin is a critical determinant of drug response. These preclinical results provided the rationale for validation of this new and innovative approach in a clinical setting.

## Materials and methods

### Cell lines and drugs

Human cancer cell lines of colorectal (HCT-8 and HT-29), head and neck (FaDu and A253) and prostate (PC3 and C42) were purchased from American type cell culture (ATCC, Manassas, VA) and maintained in RPMI 1640 with 10% fetal bovine serum (FBS). The cell lines were tested regularly using Stratogene's mycoplasma plus PCR Primer set (La Jolla, CA) and they were free from Mycoplasma. SN-38, docetaxel, 5-FU, paclitaxel, oxaliplatin, lithium chloride (LiCl) and cycloheximide (CHX) were purchased from Sigma Aldrich (St. Louis, MO). MSeA (CH_3_SeO_2_H) was purchased from PharmaSe Inc. (Lubbock, TX). Toptecan was obtained from GlaxoSmithKline (Durham, NC). Puromycin dihydrochloride, plasmid transfection medium and transfection reagents were purchased from Santa Cruz biotechnology Inc. (Santa Cruz, CA).

### Schedules and drug doses

Cells were treated with various doses of MSeA (0.05, 0.1, 0.5, 1, 5 and 10 μM) for various times (2, 4, 6, 8, 16 and 24 h). MSeA and SN-38 or docetaxel were given alone or in sequential combination. In sequential combination, 2 h treatments with SN-38 (0.7, 1.3, 0.07 and 0.3 μM against HCT-8, HT-29, FaDu and A253 respectively) or docetaxel (2 nM against PC3 and C42) started 22 h after treatment with MSeA (1 or 5 μM). HCT-8 parental and β-catenin knockout cells were treated with SN-38 (2 h), docetaxel (2 h), paclitaxel (24 h), oxaliplatin (24 h), 5-FU (24 h) and topotecan (2 h) using various doses. The doses were for SN-38 (0.5, 1 and 5 μM), for docetaxel (0.05, 0.1 and 0.5 μM), for paclitaxel (0.5, 1 and 5 μM), for oxaliplatin (1 and 5 μM), for 5-FU (50, 100 and 500 μM) and for topotecan (0.5 and 1 μM). LiCl was applied for 24 h in multiple doses (0.5, 1, 5, 10, 20 and 25 mM) alone or in combination with MSeA (5 μM).

Cycloheximide (CHX) was applied for 5, 10, 20, 30 minutes and 24 hours at a nontoxic concentration of 100 μM alone or in combination with MSeA (5 μM). Puromycin dihydrochloride was used at concentration of 20 μM for clones' selection.

### Preparation of cytoplasm and nuclear extract

Cytoplasm and nuclear extracts were prepared as previously described [41]. Briefly, to obtain cytoplasm extract, untreated and treated HCT-8 cells were harvested and suspended in lysis buffer (0.08 M KCl, 35 mM HEPES, pH 7.4, 5 mM potassium phosphate, pH 7.4, 5 mM MgCl_2_, 25 mM CaCl_2_, 0.15 M sucrose, 2 mM PMSF, 8 mM dithiothreitol). After overnight storage at -80°C, cells were passed through a 28-gauge needle, centrifuged and the supernatant collected to represent the cytoplasm extract. The remaining pellet was re-suspended in lysis buffer, sonicated, centrifuged and the supernatant collected to represent the nuclear extract.

### Silencing the expression of β-catenin

HCT-8 cells were utilized to generate a stable transfection using small hairpin β-catenin RNA (β-catenin ShRNA) purchased from Santa Cruz Biotechnology Inc. (Santa Cruz, CA). Transfection was carried out following the manufacture's instructions. Briefly, HCT-8 cells were plated 5 × 10^5 ^cells/well (6-well plate) one day before the transfection. A well with 70-80% cells confluence was transfected with control shRNA Plasmid-A (a negative control) that encodes a scrambled shRNA sequence that does not inhibit β-catenin to generate HCT-8 scrambled control (HCT-8SC). Another well with the same cells confluence, was transfected with β-catenin shRNA plasmid DNA, a β-catenin-specific lentiviral vector plasmid to knock down expression and generate HCT-8 recombinant clones (HCT-8R). Clones of stable transfectants were selected using 20 μM of puromycin dihydrochloride. After selection, 10 individual clones were evaluated using western blots and the 2 clones (HCT-8RH7 and HCT-8RF4) that demonstrated the most effect of survivin suppression were selected for further studies.

### Western blots analyses

Western blots were performed as described previously [33] to determine the effects on the intracellular protein levels. Briefly, untreated and treated cells were collected and digested using RIPA buffer (1 M Tris, 1 M NaCl, Triton X-100 and distilled water) with fresh protease inhibitor cocktail. Protein level was measured using Bio-Rad DC protein assay and a synergy HT spectrophotometer (BioTek Instruments, Winooski, VT). Equal amount of protein (50 μg) was loaded on 4-20% SDS-PAGE. After transfer, nitrocellulose membrane was rinsed with PBS-T, blocked with 5% milk and hybridized with the selected antibody. The following primary anti-bodies were used: anti-β-catenin, anti-GSK-3β (BD Biosciences, San Jose, CA) and anti-p-GSK-3β (cell signaling technology, Danvers, MA). The following secondary antibodies were used: goat anti-mouse IgG and goat anti-rabbit IgG (Santa Cruz Biotechnology, Santa Cruz, CA). After incubation with the primary and secondary antibodies, membrane was rinsed and incubated with chemilluminescence or enhanced chemilluminescence and developed using Kodak X-OMAT 2000A (First Source Inc., Rochester, NY). Anti-β-actin (Sigma Aldrich, St. Louis, MO) was used as loading controls.

### Cell growth assay

Cell growth was evaluated using sulforhodlamine B (SRB) assay as previously described and performed [33]. Briefly, after drug treatment, HCT-8WT, HCT-8SC and HCT-8RH7 and HCT-8RF4 cells were incubated in a drug-free medium for 5 days, fixed, washed and stained with SRB dye. The optical density of bound dye was measured at 570 nm using synergy HT multi-mode microplate reader (BioTek Instruments, Winooski, VT).

### Statistical analyses

Each experiment has been repeated at least 3 times. Values were presented as the mean plus or minus standard deviation. Statistical analyses were performed comparing all treatments groups using unpaired t-student test. Significant difference between groups was noted when the p value was less than 0.05.

## Competing interests

The authors declare that they have no competing interests.

## Authors' contributions

MSS performed and designed experiments, prepared and wrote the manuscript. DRR performed cytotoxicity experiments. YMR participated in study design, data interpretation and preparation of the manuscript. RGA designed the research strategy, supervised the project, assisted in data generation, results interpretation and correction of the manuscript. All authors read and approved the final manuscript.

## References

[B1] Giles RH van Es JH Clevers H Caught up in a Wnt storm: Wnt signaling in cancer Biochim Biophys Acta 2003 1653 1 24 12781368 10.1016/s0304-419x(03)00005-2

[B2] Yang F Zeng Q Yu G Li S Wang CY Wnt/beta-catenin signaling inhibits death receptor-mediated apoptosis and promotes invasive growth of HNSCC Cell Signal 2006 18 679 687 10.1016/j.cellsig.2005.06.015 16084063

[B3] Goto M Mitra RS Liu M Lee J Henson BS Carey T Bradford C Prince M Wang CY Fearon ER D'Silva NJ Rap1 stabilizes beta-catenin and enhances beta-catenin-dependent transcription and invasion in squamous cell carcinoma of the head and neck Clin Cancer Res 2010 16 65 76 10.1158/1078-0432.CCR-09-1122 20028760 PMC2844500

[B4] Tsai YP Yang MH Huang CH Chang SY Chen PM Liu CJ Teng SC Wu KJ Interaction between HSP60 and beta-catenin promotes metastasis Carcinogenesis 2009 30 1049 1057 10.1093/carcin/bgp087 19369584

[B5] Gerstein AV Almeida TA Zhao G Chess E Shih Ie M Buhler K Pienta K Rubin MA Vessella R Papadopoulos N APC/CTNNB1 (beta-catenin) pathway alterations in human prostate cancers Genes Chromosomes Cancer 2002 34 9 16 10.1002/gcc.10037 11921277

[B6] Chesire DR Ewing CM Sauvageot J Bova GS Isaacs WB Detection and analysis of beta-catenin mutations in prostate cancer Prostate 2000 45 323 334 10.1002/1097-0045(20001201)45:4<323::AID-PROS7>3.0.CO;2-W 11102958

[B7] Fevr T Robine S Louvard D Huelsken J Wnt/beta-catenin is essential for intestinal homeostasis and maintenance of intestinal stem cells Mol Cell Biol 2007 27 7551 7559 10.1128/MCB.01034-07 17785439 PMC2169070

[B8] Morin PJ Sparks AB Korinek V Barker N Clevers H Vogelstein B Kinzler KW Activation of beta-catenin-Tcf signaling in colon cancer by mutations in beta-catenin or APC Science 1997 275 1787 1790 10.1126/science.275.5307.1787 9065402

[B9] Wagenaar RA Crawford HC Matrisian LM Stabilized beta-catenin immortalizes colonic epithelial cells Cancer Res 2001 61 2097 2104 11280772

[B10] Sparks AB Morin PJ Vogelstein B Kinzler KW Mutational analysis of the APC/beta-catenin/Tcf pathway in colorectal cancer Cancer Res 1998 58 1130 1134 9515795

[B11] Conacci-Sorrell M Zhurinsky J Ben-Ze'ev A The cadherin-catenin adhesion system in signaling and cancer J Clin Invest 2002 109 987 991 11956233 10.1172/JCI15429 PMC150951

[B12] Behrens J Jerchow BA Wurtele M Grimm J Asbrand C Wirtz R Kuhl M Wedlich D Birchmeier W Functional interaction of an axin homolog, conductin, with beta-catenin, APC, and GSK3beta Science 1998 280 596 599 10.1126/science.280.5363.596 9554852

[B13] Hart MJ de los Santos R Albert IN Rubinfeld B Polakis P Downregulation of beta-catenin by human Axin and its association with the APC tumor suppressor, beta-catenin and GSK3 beta Curr Biol 1998 8 573 581 10.1016/S0960-9822(98)70226-X 9601641

[B14] Ikeda S Kishida S Yamamoto H Murai H Koyama S Kikuchi A Axin, a negative regulator of the Wnt signaling pathway, forms a complex with GSK-3beta and beta-catenin and promotes GSK-3beta-dependent phosphorylation of beta-catenin Embo J 1998 17 1371 1384 10.1093/emboj/17.5.1371 9482734 PMC1170485

[B15] Yost C Torres M Miller JR Huang E Kimelman D Moon RT The axis-inducing activity, stability, and subcellular distribution of beta-catenin is regulated in Xenopus embryos by glycogen synthase kinase 3 Genes Dev 1996 10 1443 1454 10.1101/gad.10.12.1443 8666229

[B16] De Sarno P Li X Jope RS Regulation of Akt and glycogen synthase kinase-3 beta phosphorylation by sodium valproate and lithium Neuropharmacology 2002 43 1158 1164 10.1016/S0028-3908(02)00215-0 12504922

[B17] Beurel E Kornprobst M Blivet-Van Eggelpoel MJ Ruiz-Ruiz C Cadoret A Capeau J Desbois-Mouthon C GSK-3beta inhibition by lithium confers resistance to chemotherapy-induced apoptosis through the repression of CD95 (Fas/APO-1) expression Exp Cell Res 2004 300 354 364 10.1016/j.yexcr.2004.08.001 15475000

[B18] Cliffe A Hamada F Bienz M A role of Dishevelled in relocating Axin to the plasma membrane during wingless signaling Curr Biol 2003 13 960 966 10.1016/S0960-9822(03)00370-1 12781135

[B19] Nusse R Cell biology: relays at the membrane Nature 2005 438 747 749 10.1038/438747a 16340998

[B20] Huber O Korn R McLaughlin J Ohsugi M Herrmann BG Kemler R Nuclear localization of beta-catenin by interaction with transcription factor LEF-1 Mech Dev 1996 59 3 10 10.1016/0925-4773(96)00597-7 8892228

[B21] Molenaar M van de Wetering M Oosterwegel M Peterson-Maduro J Godsave S Korinek V Roose J Destree O Clevers H XTcf-3 transcription factor mediates beta-catenin-induced axis formation in Xenopus embryos Cell 1996 86 391 399 10.1016/S0092-8674(00)80112-9 8756721

[B22] Shtutman M Zhurinsky J Simcha I Albanese C D'Amico M Pestell R Ben-Ze'ev A The cyclin D1 gene is a target of the beta-catenin/LEF-1 pathway Proc Natl Acad Sci USA 1999 96 5522 5527 10.1073/pnas.96.10.5522 10318916 PMC21892

[B23] Tetsu O McCormick F Beta-catenin regulates expression of cyclin D1 in colon carcinoma cells Nature 1999 398 422 426 10.1038/18884 10201372

[B24] He TC Sparks AB Rago C Hermeking H Zawel L da Costa LT Morin PJ Vogelstein B Kinzler KW Identification of c-MYC as a target of the APC pathway Science 1998 281 1509 1512 10.1126/science.281.5382.1509 9727977

[B25] Park WS Oh RR Park JY Kim PJ Shin MS Lee JH Kim HS Lee SH Kim SY Park YG An WG Jang JJ Yoo NJ Lee JY Nuclear localization of beta-catenin is an important prognostic factor in hepatoblastoma J Pathol 2001 193 483 490 10.1002/1096-9896(2000)9999:9999<::AID-PATH804>3.0.CO;2-R 11276007

[B26] Malanchi I Peinado H Kassen D Hussenet T Metzger D Chambon P Huber M Hohl D Cano A Birchmeier W Huelsken J Cutaneous cancer stem cell maintenance is dependent on beta-catenin signalling Nature 2008 452 650 653 10.1038/nature06835 18385740

[B27] Reya T Clevers H Wnt signalling in stem cells and cancer Nature 2005 434 843 850 10.1038/nature03319 15829953

[B28] Saltz LB Douillard JY Pirotta N Alakl M Gruia G Awad L Elfring GL Locker PK Miller LL Irinotecan plus fluorouracil/leucovorin for metastatic colorectal cancer: a new survival standard Oncologist 2001 6 81 91 10.1634/theoncologist.6-1-81 11161231

[B29] de Gramont A Figer A Seymour M Homerin M Hmissi A Cassidy J Boni C Cortes-Funes H Cervantes A Freyer G Papamichael D Le Bail N Louvet C Hendler D de Braud F Wilson C Morvan F Bonetti A Leucovorin and fluorouracil with or without oxaliplatin as first-line treatment in advanced colorectal cancer J Clin Oncol 2000 18 2938 2947 10944126 10.1200/JCO.2000.18.16.2938

[B30] Berthold DR Pond GR Soban F de Wit R Eisenberger M Tannock IF Docetaxel plus prednisone or mitoxantrone plus prednisone for advanced prostate cancer: updated survival in the TAX 327 study J Clin Oncol 2008 26 242 245 10.1200/JCO.2007.12.4008 18182665

[B31] Vermorken JB Remenar E van Herpen C Gorlia T Mesia R Degardin M Stewart JS Jelic S Betka J Preiss JH van den Weyngaert D Awada A Cupissol D Kienzer HR Rey A Desaunois I Bernier J Lefebvre JL Cisplatin, fluorouracil, and docetaxel in unresectable head and neck cancer N Engl J Med 2007 357 1695 1704 10.1056/NEJMoa071028 17960012

[B32] Cao S Durrani FA Rustum YM Selective modulation of the therapeutic efficacy of anticancer drugs by selenium containing compounds against human tumor xenografts Clin Cancer Res 2004 10 2561 2569 10.1158/1078-0432.CCR-03-0268 15073137

[B33] Azrak RG Frank CL Ling X Slocum HK Li F Foster BA Rustum YM The mechanism of methylselenocysteine and docetaxel synergistic activity in prostate cancer cells Mol Cancer Ther 2006 5 2540 2548 10.1158/1535-7163.MCT-05-0546 17041098 PMC2826137

[B34] Combs GF Jr Gray WP Chemopreventive agents: selenium Pharmacol Ther 1998 79 179 192 10.1016/S0163-7258(98)00014-X 9776375

[B35] Goldhaber SB Trace element risk assessment: essentiality vs. toxicity Regul Toxicol Pharmacol 2003 38 232 242 10.1016/S0273-2300(02)00020-X 14550763

[B36] Thomson CD Assessment of requirements for selenium and adequacy of selenium status: a review Eur J Clin Nutr 2004 58 391 402 10.1038/sj.ejcn.1601800 14985676

[B37] Schneider-Poetsch T Ju J Eyler DE Dang Y Bhat S Merrick WC Green R Shen B Liu JO Inhibition of eukaryotic translation elongation by cycloheximide and lactimidomycin Nat Chem Biol 2010 6 209 217 10.1038/nchembio.304 20118940 PMC2831214

[B38] Stambolic V Ruel L Woodgett JR Lithium inhibits glycogen synthase kinase-3 activity and mimics wingless signalling in intact cells Curr Biol 1996 6 1664 1668 10.1016/S0960-9822(02)70790-2 8994831

[B39] Zhang W Yan S Liu M Zhang G Yang S He S Bai J Quan L Zhu H Dong Y Xu N β-catenin/TCF pathway plays a vital role in selenium induced-growth inhibition and apoptosis in esophageal squamous cell carcinoma (ESCC) cells J canlet 2010 296 113 122 10.1016/j.canlet.2010.04.001 20457486

[B40] Chintala S Toth K Cao S Durrani FA Vaughan MM Jensen RL Rustum YM Se-methylselenocysteine sensitize hypoxic tumor cells to irinotecan by targeting hypoxia-inducible factor 1alpha Cancer Chemother Pharmacol 2010 66 899 911 10.1007/s00280-009-1238-8 20066420 PMC2916970

[B41] Li Y Wang Z Kong D Murthy S Dou QP Sheng S Reddy GP Sarkar FH Regulation of FOXO3a/beta-catenin/GSK-3beta signaling by 3,3'-diindolylmethane contributes to inhibition of cell proliferation and induction of apoptosis in prostate cancer cells J Biol Chem 2007 282 21542 21550 10.1074/jbc.M701978200 17522055

